# Gap junctions in olfactory neurons modulate olfactory sensitivity

**DOI:** 10.1186/1471-2202-11-108

**Published:** 2010-08-27

**Authors:** Chunbo Zhang

**Affiliations:** 1 Department of Biological, Chemical and Physical Sciences, Illinois Institute of Technology, Chicago, IL 60616, USA

## Abstract

**Background:**

One of the fundamental questions in olfaction is whether olfactory receptor neurons (ORNs) behave as independent entities within the olfactory epithelium. On the basis that mature ORNs express multiple connexins, I postulated that gap junctional communication modulates olfactory responses in the periphery and that disruption of gap junctions in ORNs reduces olfactory sensitivity. The data collected from characterizing connexin 43 (Cx43) dominant negative transgenic mice OlfDNCX, and from calcium imaging of wild type mice (WT) support my hypothesis.

**Results:**

I generated OlfDNCX mice that express a dominant negative Cx43 protein, Cx43/β-gal, in mature ORNs to inactivate gap junctions and hemichannels composed of Cx43 or other structurally related connexins. Characterization of OlfDNCX revealed that Cx43/β-gal was exclusively expressed in areas where mature ORNs resided. Real time quantitative PCR indicated that cellular machineries of OlfDNCX were normal in comparison to WT. Electroolfactogram recordings showed decreased olfactory responses to octaldehyde, heptaldehyde and acetyl acetate in OlfDNCX compared to WT. Octaldehyde-elicited glomerular activity in the olfactory bulb, measured according to odor-elicited *c-fos *mRNA upregulation in juxtaglomerular cells, was confined to smaller areas of the glomerular layer in OlfDNCX compared to WT. In WT mice, octaldehyde sensitive neurons exhibited reduced response magnitudes after application of gap junction uncoupling reagents and the effects were specific to subsets of neurons.

**Conclusions:**

My study has demonstrated that altered assembly of Cx43 or structurally related connexins in ORNs modulates olfactory responses and changes olfactory activation maps in the olfactory bulb. Furthermore, pharmacologically uncoupling of gap junctions reduces olfactory activity in subsets of ORNs. These data suggest that gap junctional communication or hemichannel activity plays a critical role in maintaining olfactory sensitivity and odor perception.

## Background

Research over the past two decades has greatly enhanced our understanding of the mechanisms by which olfactory receptor neurons (ORNs) detect and transmit odor information [1,2]. However, little is known if odor-elicited activity in the primary olfactory pathway is modulated before transmission to the olfactory bulb. It is evident that ORNs expressing the same receptor may transmit different patterns of signals to the same glomerulus under certain conditions. *In vivo *glomerular imaging studies show that the dynamic range of ORN input into a defined glomerulus is larger than that reported for single isolated ORNs [3,4]. Subsequent studies further demonstrate that ORN input is responsible for, or partially responsible for, the diverse spatial and temporal dynamics observed in glomeruli [5,6]. These results are in sharp contrast to those from single isolated ORNs in which the dose-response relationships are typically saturated within a log unit [7,8]. The difference appears irrelevant to the techniques used. For example, the range of dose-response relationship was broad when patch recorded from a neuron situated in a sheet of olfactory epithelium [9,10] or when single unit recordings were performed in the olfactory turbinates [11]. Clearly, some important pieces are missing. In this study, I present data to demonstrate that impairment of gap junctions or altered assembly of gap junctions or hemichannels in ORNs modulates odor responsiveness and leads to changes in olfactory activation patterns in the olfactory bulb. My study suggests that coordination of neuronal activity between ORNs through gap junctions or hemichannels may constitute an important mechanism in modulating olfactory sensitivity and odor perception.

Connexins are gap junction forming subunits that assemble into hexametric connexons or hemichannels. Two hemichannels, contributed by adjacent cells, are docked at the extracellular loops to form intercellular gap junctional channels. These channels allow electronic coupling and/or passage of small molecules, including ions, nutrients, metabolites or second messengers between coupled cells [12]. Gap junctional coupling can profoundly change electrical activity in ORNs since these neurons possess high input resistance. Indeed, in ORNs the opening of one channel can induce generation of an action potential [13,14]. Since one gap junctional channel formed by connexin 43 (Cx43) could transfer about 50% of the steady state current generated by one cell to its neighbor [15], a few gap junctional channels could substantially alter the electrical properties of ORNs.

Gap junctions in neurons are critical for maintaining physiological activity in sensory systems. In mice, deletion of connexin 36 (Cx36), connexin 45 (Cx45) or connexin 57 (Cx57) reduces response acuity or impairs sensory transduction in the vision system [16-19]. In the olfactory bulb, Cx36 mediated gap junctional coupling contributes to mitral cell lateral excitation [20]. Electronic coupling among mitral cells allows temporally coherent output from an odor-specific glomerular unit [21,22]. Furthermore, a report from the same group shows that Cx36 is involved in maintaining the amplitude and in generating long-lasting olfactory nerve evoked EPSP in glomeruli [6].

**Figure 1:**
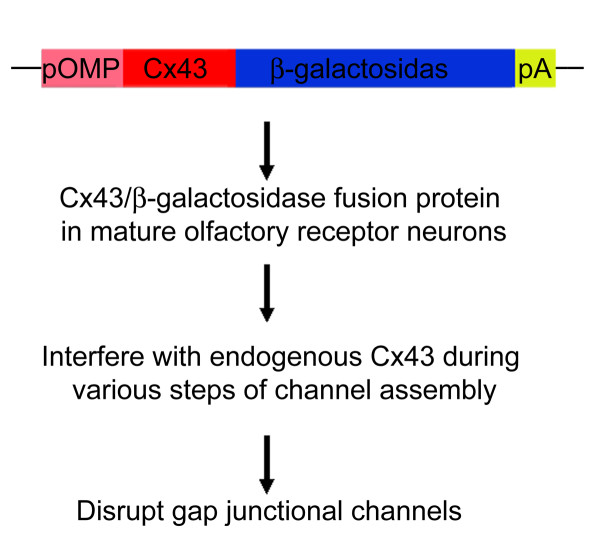
**A diagrammatic illustration of the transgene construct and expected outcomes**. In the construct, an 880 bp proximal region of the olfactory marker protein promoter (pOMP) links to the full length of connexin 43 coding sequence (Cx43) and followed by full length of sequence encoding β-galactosidase. A Poly A (pA) is attached at the end.

**Figure 2:**
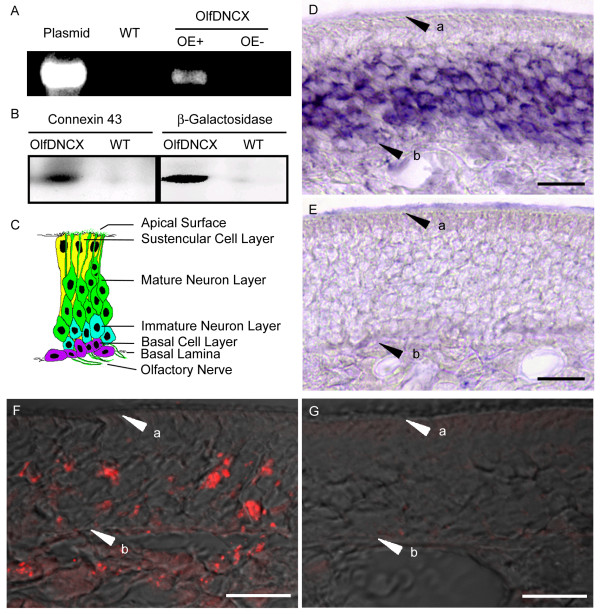
**Characterization of OlfDNCX**. **A**. The PCR results from reverse transcription. Templates used for PCR reaction were the plasmid of the construct (plasmid), cDNA of olfactory turbinates in wild type (WT) and OlfDNCX mice. In OlfDNCX, OE- is the control that was processed identically as OE+ except missing SuperScript II in reverse transcription. **B**. Western analysis of protein homogenates of olfactory turbinates revealed a protein band around 120 kD in OlfDNCX, but not in WT, that was immunoreactive to antibodies against connexin 43 and β-galactosidase. **C**. A cartoon indicates the arrangement of epithelial cells in the olfactory epithelium. **D and E**. *In situ *hybridization in the olfactory epithelium. *In situ *hybridization using the antisense β-galactosidase riboprobe showed that the signal was localized to a band in the middle of the olfactory epithelial layer (**D**). The control (**E**) was processed identically as (**D**) except that sense β-galactosidase riboprobe was used. **F and G**. Confocal images displaying immunoreactivity for β-galactosidase (red) overlaid on Nomarski images. Immunostaining was observed in OlfDNCX (**F**), but not in WT (**G**). Arrowheads point to a, apical surface; and b, basal lamina. Bar = 20 μm.

Our studies have demonstrated expression of multiple connexins (36, 43 and 45, 57) in ORNs in addition to their expression in the olfactory bulb [23-26]. These connexins are heterogeneously distributed within the olfactory epithelium in regional and partially overlapping patterns. We postulate that gap junctional coupling between ORNs, or between ORNs and sustentacular cells, plays a role in modulating olfactory neuronal activity. However, in a follow-up freeze-fracture immunocytochemical study to visualize gap junctions in the olfactory epithelium and olfactory bulb, we did not identify gap junction plaques in ORNs [27]. This result diverges from our earlier studies where we used a combination of approaches including utilizing transgenic mice [23,24]. Even though the negative results from freeze-facture studies do not establish an absence of gap junctions in ORNs due to its technical nature, it certainly casts doubts on presence of gap junctions in ORNs. One probable explanation for this discrepancy is that the number of gap junctional channels in ORNs is sparse and cannot be identified by freeze-fracture studies since non-clustered gap junctional channels would not form typical gap junction plaques. However, a few sparsely distributed gap junctions, if present in ORNs, could profoundly modulate olfactory coding. To further address the possible involvement of gap junctions in olfactory coding, I used a dominant negative transgenic approach to *specifically *disrupt gap junctions in mature ORNs while gap junctions in sustentacular cells and basal cells in the olfactory epithelium and gap junctions in other tissues remain intact. The dominant negative transgenic mouse OlfDNCX expresses an olfactory marker protein (OMP) promoter driven dominant negative variant Cx43/β-galactosidase fusion protein (Cx43/β-gal) in mature ORNs with minimal expression in sustentacular cells, basal cells and immature ORNs (Figure 1 and 2). This fusion protein has a β-gal reporter protein directly fused to the C-terminus of Cx43 [28,29]. Cx43/β-gal (inactive) interferes with endogenous Cx43 during protein trafficking and thus decreases transport of Cx43 to the plasma membrane [29,30], limiting the formation of functional gap junctions [31]. Transgenic mice that express Cx43/β-gal driven by the human elongation factor-1α promoter die shortly after birth and display phenotypes typical of Cx43 knock out mice, including reduced dye coupling and heart malformation [28]. This indicates that *in vivo *expression of Cx43/β-gal can powerfully inhibit gap junctions. Using OlfDNCX mice, I demonstrate that gap junctional communication in the olfactory epithelium modulates olfactory activity at the peripheral level and alters glomerular activation patterns (odor maps) in the olfactory bulb. Topological changes in odor maps due to gap junctional modulation could affect perception of odor quality or quantity.

## Results

### Expression of Cx43/β-gal transcript and protein in the olfactory epithelium

I first used the reverse transcription PCR (RT-PCR) method to investigate expression of Cx43/β-gal in olfactory turbinates. I collected total RNA of olfactory turbinates from OlfDNCX mice and their wild type (WT) littermates. PCR amplification of reverse transcription products using a primer pair spanning the regions encoding for the two proteins fused in the construct (Cx43 and β-gal) showed a single band at the estimated size in OlfDNCX but not in WT (Figure 2A). An identical sample processed without addition of reverse transcriptase did not yield a product (OE- in Figure 2A). The RT-PCR product from olfactory turbinates was subcloned and sequenced confirming amplification of the region bridging nucleotides coding for the C-terminus of Cx43 and the beginning of the β-gal coding sequence.

To verify presence of the Cx43/β-gal protein in the olfactory epithelium, I performed western immunoblot analysis and immunohistochemistry in adult mouse turbinates. Western analysis using either a mouse monoclonal antibody against Cx43 or a rabbit polyclonal antibody against β-gal revealed a protein band with molecular weight of approximately 120 kDa in OlfDNCX but not in WT (Figure 2B).

### Cellular localization of Cx43/β-gal mRNA and protein in the olfactory epithelium

The olfactory epithelium is made up of three cell types: ORNs, sustentacular cells, and basal cells (Figure 2C). About 80% of the olfactory epithelial cells are neurons and they are often found in columns with their cell bodies in close contact with each other and dendrites surrounding the nucleated portion of sustentacular cells [32,33]. Cells adjacent to mature olfactory neurons are mature olfactory neurons, immature olfactory neurons and sustentacular cells. To determine expression patterns of mRNA for Cx43/β-gal, *in situ *hybridization was carried out in multiple coronal sections spanning the entire olfactory epithelium. The hybridization signal with antisense β-gal cRNA was observed in a band located in the middle of the olfactory epithelial layer (Figure 2D), a pattern similar to expression of endogenous OMP mRNA that is expressed exclusively in mature ORNs (not shown). Sense β-gal cRNA was hybridized under identical conditions yielding a faint background signal (Figure 2E).

Figure 2F shows the localization of β-gal immunoreactivity in the olfactory epithelium. Immunofluorescence was observed in cell bodies situated in a medial band of the olfactory epithelial layer but not at the apical layer of the olfactory epithelium. There was also labeling in the region of axon bundles underneath the olfactory epithelium, which shows robust labeling for endogenous Cx43 [23]. This pattern is consistent with expression of the Cx43/β-gal protein in mature ORNs. In contrast, immunolabeling was not detected in the olfactory epithelium of WT mice (Figure 2G). Interestingly, immunolabeling for Cx43/β-gal did not show the characteristic punctate pattern of immunoreactivity of endogenous Cx43 observed in normal mice [23], but rather showed relatively large aggregates of immunofluorescence, presumably reflecting clustering of misassembled connexins, consistent with reports by others [31]. Similar results were obtained when another antibody, polyclonal guinea pig antibody against β-gal, was used for immunohistochemistry (data not shown). No noticeable gross morphological differences were observed between the epithelium of OlfDNCX mice and WT.

### Introduction of the transgene does not alter expression of marker genes or other connexins in the olfactory epithelium

I further used the real time quantitative PCR (qPCR) method to investigate whether expression of Cx43/β-gal induced organizational changes of the olfactory epithelium. I compared expression of a few marker genes of the olfactory epithelium and connexins expressed in the olfactory epithelium between OlfDNCX and WT using qPCR. qPCR is a sensitive method in monitoring cellular changes by quantitative measurement of gene expression. Total RNA of the olfactory epithelium from 17 individuals was collected under identical conditions and used for qPCR analysis. The primers used in the study are listed in Table 1. The housekeeping gene glyceraldehyde-3-phosphate dehydrogenase (GAPDH) was used as the internal control and the data were presented as comparative threshold cycle (Ct) (ΔΔCt) values (Table 2). Among the examined genes, OMP, GAP43 and CYP2A5 are selectively or predominantly expressed in mature ORNs, immature ORNs and sustentacular cells, respectively [34-37]. G_olf _is a G_s_α-like G protein that is involved in the first step of olfactory signal transduction cascade [38,39], and I7 is among a few olfactory receptors whose ligands are characterized [40,41]. In addition, I measured expression of Cx36, Cx43, Cx45 and Cx57 to see whether OlfDNCX exhibited altered expression of endogenous connexins. These connexins are expressed in the olfactory epithelium [23-26]. Measurement of endogenous Cx43 transcription is possible in OlfDNCX using specific primers recognizing Cx43 3' untranslated region (Cx43-3U) since the transgene does not contain this portion of the gene. Table 2 shows that none of the I7 receptor, OMP, GAP43, G_olf _or CYP2A5 mRNA levels was different between OlfDNCX and WT after they were normalized to the housekeeping gene GAPDH. These data, in addition to the electrophysiological evidence shown below, suggest that expression of Cx43/β-gal in the ORNs does not have a significant effect on olfactory signal transduction machinery, the life span of a specific olfactory receptor, the population of mature and immature olfactory neurons, and the population of sustentacular cells. Table 2 also demonstrates that Cx43/β-gal did not alter expression of listed endogenous connexins either. This result correlates with a previous report that expression of Cx43/β-gal did not stimulate expression of endogenous Cx43 even though Cx43 assembly was affected [42].

**Table 1:** Primers used in real time quantitative PCR

Genes	Primer pairs
Glyceraldehyde-3-phosphate dehydrogenase (*GAPDH*)	Forward: GTGGACCTCATGGCCTACAT Reverse: TGTGAGGGAGATGCTCAGTG
Olfactory marker protein (*OMP*)	Forward: CCGCCGCCATCTTCTG Reverse: CGTCTGCCTCATTCCAATCC
GTP-binding protein Golf alpha subunit (*Golf*)	Forward: TTTGGGCAACAGCAGCAA Reverse: CTCGCGGCGTCCTTTTTC
Growth associated protein 43 (*Gap43*)	Forward: ACCACCATGCTGTGCTGTATG Reverse: TCAATCTTTTGGTCCTCATCATTC
Cytochrome P450, family 2, subfamily A (*CYP2A*)	Forward: GCTGGGAAGCTTCCAGTTCAC Reverse: GGCCCTGCAGCTCCTTAAA
I7 olfactory receptor (*I7*)	Forward: TAAGGCACTCTCAGCTTTTGACA Reverse: GAGTGCGACGT AGGGCTTT
Connexin 36 (*Cx36*)	Forward: GAGGTTAAAGAGCTGACTCCACATC Reverse: TCGGAGCTTGGACCTTGCT
Connexin 43, 3' untranslated region (*Cx43-3U*)	Forward: AAAGATTGCCCATGTATTTGCA Reverse: GACACAAAGGTGGGACAGATTTG
Connexin 45 (*Cx45*)	Forward: ACAGTGTTCCCAGGCACATG Reverse: CTGGAAGACACAACCTGAAAGTTCT
Connexin 57 (*Cx57*)	Forward: GAAGTCGCAAGGCCAGCTT Reverse: CACTATGCCGTTGTCCCTTTTC

**Table 2:** Comparative Ct (ΔΔCt) values for genes examined (n = 17)

Genes	OlfDNCX	Wild type
*OMP*	2.14 ± 0.17	1.92 ± 0.13
*Golf*	1.98 ± 0.21	1.88 ± 0.12
*Gap43*	0.65 ± 0.01	0.70 ± 0.05
*CYP2A*	1.43 ± 0.08	1.31 ± 0.06
*I7*	3.97 ± 0.29	3.17 ± 0.35
*Cx36*	1.73 ± 0.20	2.36 ± 0.32
*Cx43-3U*	1.01 ± 0.08	1.02 ± 0.03
*Cx45*	0.92 ± 0.05	0.96 ± 0.02
*Cx57*	0.91 ± 0.15	1.03 ± 0.05

### OlfDNCX mice display altered electrophysiological responses to octaldehyde in ventral areas of the epithelium

Underwater electroolfactogram (EOG) recording was used to examine whether electrophysiological responses to odors were different between OlfDNCX mice and WT. EOG records the field potential generated by populations of ORNs in response to stimuli [43] and is a reliable method to test olfactory responses to odors in various animals, including rodents [44]. First, I applied 500 μM 3-isobutyl-1-methylxanthine (IBMX), a phosphodiesterase inhibitor that elicits responses by increasing intracellular cAMP. This drug consistently induced large olfactory responses where the magnitudes did not differ between WT and OlfDNCX in various locations (not shown), suggesting that Cx43/β-gal expression in the olfactory epithelium of OlfDNCX did not result in a gross interference of the signal transduction machinery in ORNs.

**Figure 3:**
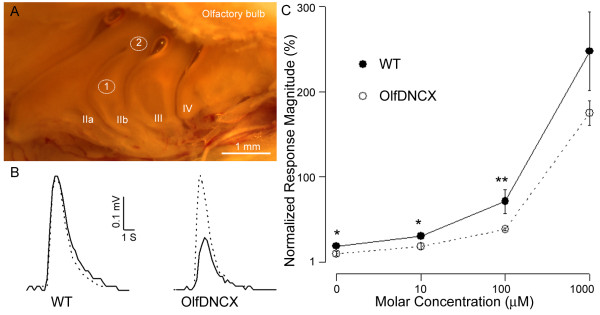
**OlfDNCX display altered responses to odorants**. **A**. A photograph of the turbinates in a mouse showing EOG recording sites. Endoturbinates are indicated by roman ordinals. The approximate locations for EOG recordings are indicated. **B**. Typical EOG responses for octaldehyde (solid line) and benzaldehyde (dotted line) in wild type mice (WT) and in OlfDNCX in Position 1. Duration of the stimulation and the response magnitude are as indicated. **C**. Dose-response relationships for peak EOG responses to octaldehyde (normalized to responses of 100 μM benzaldehyde) in OlfDNCX (open circle) and their WT littermates (solid circle). Significant differences between the two groups are indicated by asterisks (*, p < 0.05; **, p < 0.01; n = 11).

Because I suspected that modulation of odor responses by gap junctions would be of a relatively small magnitude, and because there was a large variation in the absolute magnitude of the EOG responses to an odorant from mouse to mouse, I measured the response to one odorant normalized to the response of another odorant (normalized response), as has been routinely done in EOG recordings in rat [44]. EOG recordings were conducted at ventral (Position 1) and dorsal (Position 2) positions (indicated in Figure 3A) because an earlier study shows that Cx43 is more abundantly expressed in Position 1 than in Position 2 [23]. I started with stimulation of benzaldehyde, 1,8-cineole, and octaldehyde at the concentration of 100 μM. It appeared that olfactory responses to octaldehyde were consistently lower in OlfDNCX, compared to WT, when recorded from Position 1 (Figure 3B). Indeed, when I quantified the response magnitudes of octaldehyde by normalizing to those of benzaldehyde, the normalized responses were significantly different between OlfDNCX and WT (p < 0.0001) (Figure 3B, Table 3). The ratios of cineole to benzaldehyde did not differ between OlfDNCX and WT in Position 1 (Table 3). In contrast, no significant differences were found between OlfDNCX and WT for EOG responses recorded from Position 2, an area expressing limited Cx43. In the statistical analysis, p values were adjusted to correct for multiple comparisons by using the false discovery rate procedure [45]. Table 4 lists normalized olfactory response magnitudes to 2,5-dimethyl pyrazine, ethyl acetate and heptaldehyde recorded from Position 1. The olfactory responses of ethyl acetate and heptaldehyde were lower in OlfDNCX compared to WT, when they were normalized to those of benzaldehyde.

**Table 3:** Comparison of olfactory response magnitudes between OlfDNCX and their wild type littermates

Responses (normalized to benzaldehyde)	Mouse type	**Position 1**^**a**^		Position 2	
		
		Mean ± SE	n	Mean ± SE	n
Octaldehyde	OlfDNCX	0.717 ± 0.040**^b^	25	0.998 ± 0.058	19
	Wild type	1.115 ± 0.062		1.015 ± 0.170	

Cineole	OlfDNCX	1.567 ± 0.126	19	0.727 ± 0.057	15
	Wild type	1.730 ± 0.197		0.919 ± 0.170	

**a**, Electroolfactogram (EOG) recording positions 1 and 2 are as indicated in Figure 3A.

**b**, ** indicates highly different between OlfDNCX and wild type (p < 0.01).

**n**, sample size.

**Table 4:** Comparison of olfactory response magnitudes between OlfDNCX and their wild type littermates recorded from Position 1^a^

Responses (normalized to benzaldehyde)	Mouse type	Mean ± SE	n
2,5-Dimethyl pyrazine	OlfDNCX	0.779 ± 0.031	8
	Wild type	0.644 ± 0.061	

Ethyl Acetate	OlfDNCX	0.314 ± 0.022**^b^	16
	Wild type	0.529 ± 0.050	

Heptaldehyde	OlfDNCX	0.189 ± 0.072*	11
	Wild type	0.386 ± 0.055	

**a**, Electroolfactogram (EOG) recording Position 1 is as indicated in Figure 3A.

**b**, * indicates significantly different between OlfDNCX and wild type (p < 0.05) and ** indicates highly different (p < 0.01).

**n**, sample size.

I further compared olfactory responses to octaldehyde between OlfDNCX and WT at various concentrations in Position 1. Figure 3C shows dose-response relationships of EOG responses to octaldehyde, when normalized to 100 μM benzaldehyde. The normalized response magnitudes were lower for OlfDNCX in the concentration range of 1-100 μM, indicating the differences of olfactory responses between OlfDNCX and WT are significant at low to moderate concentrations of stimuli. EOG responses for OlfDNCX at higher concentrations (1 mM, Figure 3C, and 10 mM not shown) were smaller in magnitude than the responses for WT, but due to high inter-animal variability the values were not significantly different.

### Calcium imaging reveals that octaldehyde-responsive neurons are susceptible to gap junction uncoupling reagents

**Figure 4:**
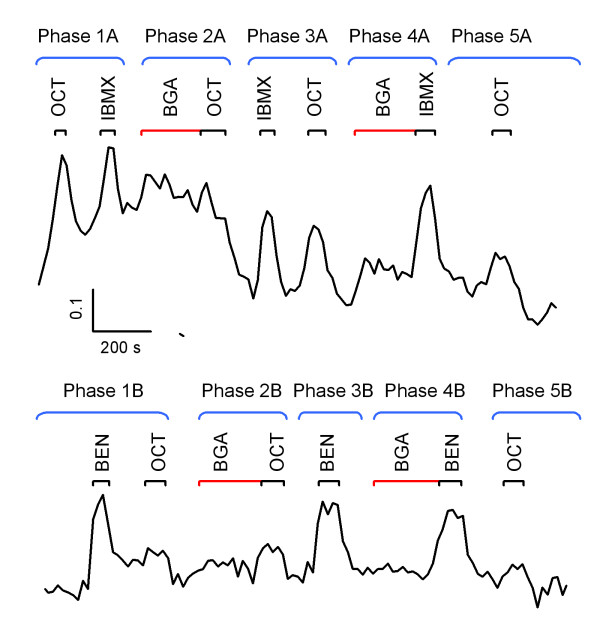
**Gap junction coupling is neuron specific**. Raw data of calcium imaging showing representative neuronal responses to stimuli in a period over 30 min. Data were binned every four frames after recordings using the Excel program and presented in the ratio of F340/F380. For the convenience of description, data are grouped into 5 phases. Duration of stimulation is indicated by black (for odorants) or red (for BGA) bars on the top. BEN, 100 μM benzaldehyde; BGA, 1 μM 18β-glycyrrhetinic acid, a gap junction uncoupling reagent; IBMX, 500 μM 3-isobutyl-1-methylxanthine; OCT, 100 μM octaldehyde.

I used calcium imaging to monitor individual neuronal responses with or without the influence of gap junction uncoupling reagents in WT mice. Calcium imaging was conducted on the surface of the epithelium in intact turbinates as shown in Figure 3A. Because the recording ORNs were situated within an intact olfactory epithelium, they were under optimal biological conditions they could possibly have in an *in situ *preparation. This *in situ *preparation allowed me to study cell-to-cell communication under physiological conditions. Figure 4 shows examples of individual ORNs responding to odorants before and after application of the gap junction uncoupling reagent 18β-glycyrrhetinic acid (BGA) at 1 μM. At this concentration, BGA itself induced negligible intracellular calcium changes (Phase 2A, 2B, 4A and 4B in Figure 4). Interestingly, at this concentration the effects of BGA to odor-evoked responses were neuron specific. Neuron A, an octaldehyde-responsive neuron, was responsive to 500 μM IBMX as well (Phase 1A). After application of 1 μM BGA, responsiveness of Neuron A to octaldehyde was reduced to threshold levels (Phase 2A). Since the effect of BGA is reversible [46,47], Neuron A regained its response magnitude to octaldehyde minutes later (Phase 3A). Phase 4A in Figure 4 shows that responsiveness of Neuron A to IBMX was not influenced by application of BGA. The same applied to its responsiveness to 76 mM KCl (high K) (data not shown). These results indicate that BGA does not interfere with normal physiological capability of Neuron A because it did not interfere with IBMX- or high K-evoked responses. However, the responses to 100 μM octaldehyde observed in Phase 1A were significantly modulated by the BGA treatment. I speculate that the olfactory receptor of Neuron A is only moderately sensitive to octaldehyde and gap junctional coupling with other neurons is necessary to sustain the response magnitude observed in Phase 1A.

Neuron B was an example of a different type of neurons. It was primarily a benzaldehyde-specific neuron since it responded well to benzaldehyde but weakly to octaldehyde. Evidently, the olfactory responses of Neuron B were relatively independent of gap junctions since application of BGA had little influences on responsiveness to benzaldehyde or to octaldehyde (see Phase 2B and 4B in Figure 4).

These results were repeatable in multiple preparations. Among 456 regions of interests (ROI) analyzed, 164 did not respond to applied odor stimuli and 12 died during imaging. In 169 octaldehyde responsive ROI, 132 of them had response patterns similar to Neuron A - application of BGA rendered octaldehyde responses to threshold levels. About 37 octaldehyde-responsive neurons were insensitive to BGA treatments. Application of 10 μM of carbenoxolone, another gap junction uncoupling reagent, resulted in the same results as BGA. None of the IBMX or high K responses was affected by gap junction uncoupling reagents. The effects of BGA were observed to be neuron dependent. As shown in Figure 4, octaldehyde responses in Neuron B were not affected by the BGA application (Phase 2B). These data indicate that for some octaldehyde-responsive neurons, coupling with other neurons is critical for maintaining olfactory response magnitudes. However, there are neurons whose actions are independent of gap junctional modulation.

### Octaldehyde-elicited odor activity maps differ between OlfDNCX and wild type mice

Upon odor stimulation, ORNs transmit information through axonal action potentials to distinct neuropil structures in the olfactory bulb called glomeruli. In addition to activating mitral cells, synaptic transmission activates juxtaglomerular cells (periglomerular cells and tufted cells) surrounding each glomerulus. Thus, the activity of surrounding juxtaglomerular cells reflects activation of a particular glomerulus. To study whether disruption of gap junctions in the olfactory epithelium affects odor presentation in the olfactory bulb, I constructed odor activity maps of octaldehyde in OlfDNCX and WT using a mapping tool developed in Restrepo laboratory [48,49]. The odor activation maps were generated by scoring odor-activated glomeruli throughout the entire glomerular layer of the olfactory bulb and then performing a series of computational analyses. Determination of an activated glomerulus was made by measurement of odor-evoked *c-fos *mRNA transcription in juxtaglomerular cells surrounding the glomerulus. More details of the odor activity mapping is described in the "Methods" section and in previous publications [48,49]

**Figure 5:**
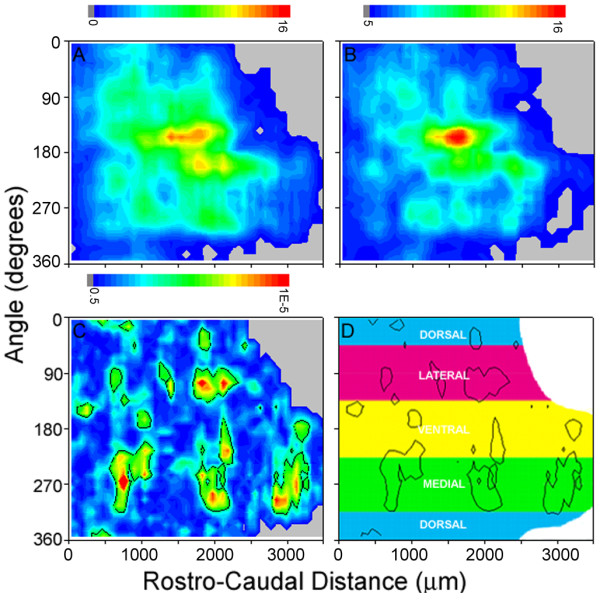
**OlfDNCX mice exhibit a reduced glomerular activity map to octaldehyde**. **A and B**. Pseudocolor contour maps showing averaged octaldehyde-evoked glomerular activation patterns in wild type mice (**A**) and their OlfDNCX littermates (**B**) (n = 8 for each map). The color denotes the number of activated glomeruli in a region spanning 216 μm in the rostrocaudal direction and 30 degrees in the angle dimension. The pseudocolor scale varies linearly from blue (0 glomerulus) to red (16 glomeruli). **C**. p values for a pixel by pixel Mann-Whitney test of differences between the odor maps in (**A**) and (**B**). Areas enclosed by black contour lines are regions that differ significantly (p < 0.006). The pseudocolor scale varies logarithmically from a p value of 0.5 (blue) to 10^-5 ^(red). **D**. A diagram indicating the locations of domains that differ between OlfDNCX and wild type mice.

When mice were exposed to fresh air passing through a vessel with 0.001% octaldehyde in odorless mineral oil, a moderate intensity stimulus known to be detectable by mice (Slotnick, Zhang and Restrepo, unpublished), odor-evoked *c-fos *mRNA expression was elevated in juxtaglomerular cells in discrete areas. Figure 5 shows the odor maps of averaged glomerular activation evoked by exposure to octaldehyde in WT (Figure 5A) and OlfDNCX mice (Figure 5B). The areas of maximal activation in the ventral zone (shown in yellow and red in the figure) overlap. However, consistent with the decreased responsiveness of the olfactory epithelium to octaldehyde in the EOG experiments, overall levels of activation were lower for OlfDNCX. In OlfDNCX, a total number of 458 ± 25.5 glomeruli were activated, while the number of glomeruli activated by octaldehyde in WT was 620 ± 59.5 (mean ± SE, n = 8 for each group, p < 0.05). In order to explore regional differences in glomerular activation, I used a point by point Mann-Whitney test with confidence interval p values corrected for multiple comparisons using the false discovery rate procedure [45,48,49]. The areas within the black contour lines of Figure 5C were statistically different. There were regions, particularly in the ventromedial, medial and caudolateral areas where the number of glomeruli activated by octaldehyde was substantially smaller for OlfDNCX compared to WT.

## Discussion

I have directly assessed the role of Cx43 in ORN activity by calcium imaging of individual neurons in an intact olfactory epithelium preparation and by characterization of OlfDNCX transgenic mice. My study has demonstrated that Cx43 is involved in modulating responsiveness of ORNs to odors in the periphery and consequently affects odor activation patterns in the olfactory bulb. The data indicate that precise assembly of connexin protein subunits in the membrane is required for maintaining response magnitudes in subsets of neurons including a group of octaldehyde-responsive neurons. Impairment of gap junctions or hemichannels by the OlfDNCX transgenic approach or by pharmacological gap junction uncoupling reduces olfactory responsiveness to octaldehyde. This is the first study showing that intercellular communication between mature ORNs and other epithelial cells (ORNs and/or sustentacular cells) or hemichannel activity has functional consequences under normal physiological conditions. Since the dominant negative Cx43/β-gal protein is expressed exclusively in mature ORNs of OlfDNCX mice with negligible influences to developmental processes and maturation, or to gap junctions in other olfactory epithelial cells, decreased EOG responses in OlfDNCX reflect the involvement of gap junctions or hemichannels in modulating olfactory activity at the peripheral level by spread of excitation to neighboring cells.

My findings cannot discern between the effects of Cx43 gap junctions as opposed to Cx43 hemichannels but the possibility remains that gap junctional coupling between ORNs exists as discussed below. In this case, ORNs would not be independent entities but rather certain subsets of ORNs form functional units in which individual activity is subject to modulation by the activity of neighboring ORNs.

### Transgenic dominant negative approach to study the functional role of gap junctions

Because of technical difficulties in measuring physiological changes directly at the systemic level and unavailability of useful pharmacological tools that could be employed to interfere specifically with gap junctional communication *in vivo*, assessment of the functional significance of gap junctions is largely based on the discovery of disease-causing mutation in human connexins and germline targeted disruptions of mouse connexin genes [50,51]. In particular, accessing the function of gap junctions in ORNs is a daunting task because gap junctions are also expressed in other olfactory epithelial cell types (basal cells and sustentacular cells) and because gap junctions are sparsely expressed in ORNs, making direct physiological assessment difficult. Generation of OlfDNCX is valuable for studying the role of gap junctions in ORN function. This dominant negative transgenic approach has advantages compared to germline targeted gene disruption, and would be complementary to conditional gene disruption. Since I used the OMP promoter to drive the transgene, the dominant negative Cx43/β-gal is expressed robustly in mature ORNs, ensuring that the largest effect of the transgene on gap junctions occurs in mature ORNs (Figure 1 and 2).

Dominant negative inhibition is achieved by producing an inhibitory variant that shares similarities in structure with the endogenous target(s). It is possible that Cx43/β-gal interacts with connexins other than Cx43 in mature ORNs. In fact, Das Sarma et al. [31] suggest that Cx43/β-gal is capable of interfering with connexin 46 (Cx46), but not with connexin 32 in the HeLa cell line, where cells have the ability to form Cx43/Cx46 heteromeric complexes. Studies have also shown that Cx45 is able to oligomerize with Cx43 [52,53]. It remains to be determined whether Cx43/β-gal oligomerizes with Cx57 because 709 nucleotides of the Cx57 N-terminal are approximately 70% homologous to that of Cx43. Our studies show that mouse olfactory epithelium expresses Cx36, Cx43, Cx45 and Cx57 [23-26]. Using Cx46 knockout mice whose Cx46 gene is replaced by the β-gal reporter [54], we find that Cx46 is not expressed in ORNs (Zhang and Kumar, unpublished). Cx43/β-gal does not affect Cx36 due to structural diversity. The inhibitory variant can potentially interfere with gap junctions of Cx45 and Cx57 in addition to Cx43 according to gene sequence homologues. Whether or not Cx43/β-gal interferes with other connexins in ORNs would not undermine my findings that gap junctional communication plays a role in modulating olfactory responses at the peripheral level, although it is desirable to determine the specific molecular interaction of dominant negative inhibition in future studies.

### Dysfunction of gap junctions alters olfactory responsiveness in the periphery

My data indicate that OlfDNCX, compared to WT, have reduced normalized EOG responses to octaldehyde, heptaldehyde and ethyl acetate, but not to cineole and 2,5-dimethyl pyrazine (Table 3 and 4). Because the EOG is elicited by the voltage drop induced by the sum of the odor-evoked currents from all ORNs in the neighborhood of the recording electrode, the EOG is an integrated response: the more ORNs that respond, the larger the voltage drop recorded in the EOG. The decreased magnitude in the EOG of OlfDNCX mice reflects decreased current flowing through the paracellular junctions. This indicates that uncoupling of ORNs by dominant negative downregulation of gap junctions results in a smaller number of ORNs responsive to a particular odor and hence a smaller EOG in OlfDNCX mice compared to WT.

The calcium imaging data presented here correlate with the notion that uncoupling of gap junctions in subsets of neurons reduces the number of ORNs responsive to a particular odor as uncoupling of gap junctions rendered responses to threshold levels in subsets of neurons (Figure 4, Neuron A). Consistent with the sparseness of immunolabeled connexin puncta in the olfactory epithelium in mice [23,24,55], not all neurons are under modulation of gap junctions. My results are in agreement with a report in *Necturus maculosus*, where Delay and Dionne [56] found that only a small fraction of olfactory neurons contained gap junctional channels. While a small number of coupled ORNs argues against a *general *role for gap junctions in modulation of ORN function, its significance in olfactory transduction should not be underestimated. Reduced olfactory responses at the peripheral pathway and altered odor maps suggest that impairment of gap junctions may have profound effects on olfactory perception.

Because neighboring ORNs in the olfactory epithelium often express different olfactory receptors [2], coupled neurons may have different responsiveness. Then, what is the significance of coupling among different ORNs? Since expression of connexins in the olfactory epithelium is less abundant than that in the retina [17,24,57] and Zhang, unpublished), coupling among different ORNs might be at low strength, which is consistent with our computer simulation based on my data presented above [58]. Low strength coupling synchronizes subthreshold or threshold activity of neurons by providing low frequencies of current flow through gap junctional channels. Under these circumstances, coupling increases olfactory sensitivity. At higher concentrations, individual ORN firing is stimulated by the cognate odors and is dependent on its receptor specificity. This mechanism would allow for increased olfactory sensitivity of odors without compromising odor quality discrimination.

Low strength coupling of ORNs indicates that coupling sites are mediated by a small number of gap junctional channels. This provides a probable reason for unsuccessful freeze-facture identification of typical gap junction plaques in ORNs within the olfactory epithelium examined [27]. Heterogeneous distribution of connexins throughout the olfactory epithelium and technical difficulties in sample preparation may be other factors for the negative results since the methodology itself prohibits it from providing a picture encompassing large areas and the freeze-fracture method is adverse to bony tissues. Small amount of gap junctional channels may be also accounted for a low incidence of dye coupling [56,59]. Ample data demonstrate that dye coupling depends on the nature of connexins, molecules of dyes, and the density of channels [60-62].

### Influence of connexin hemichannels in olfactory perception?

Recently, there has been an increase in attention to the function of hemichannels formed by connexins [63-65]. While hemichannels are inevitably present in gap junction expressing cells because gap junctions are formed after docking to newly assembled hemichannels in adjacent cells [66], their opening, which becomes prominent under pathological conditions, could result in release of a series of biologically relevant signaling molecules such as ATP, glutamate, glutathione, NAD^+^, and prostaglandin E2 [65]. Hemichannels made by Cx43 or structurally related connexins, if presented in mature ORNs, will be impaired in OlfDNCX. My calcium imaging data presented here (Figure 4) suggest presence of gap junctional coupling in ORNs because application of gap junction uncoupling reagents reduced intracellular [Ca^2+^]. Alternatively, blocking of hemichannels with BGA or carbenoxolone would have led to opposite results.

Under normal physiological conditions, the newly formed hemichannels are in closed states before forming gap junctions. An elegant dual-patch recording showed that in neonatal rat heart cells channel opening followed formation of gap junctions [67]. Studies in the mammalian cell lines and *Xenopus *oocytes demonstrate that opening of hemichannels made by various connexins requires strong depolarization (above 0 mV) or removal of extracellular calcium (ref: Spray et al. [63]). Subthreshold cellular insults or acute pathological threatening conditions may also lead to opening of hemichannels [65,68-71]. On the other hand, physiological significance of neurotransmitter release through hemichannels in retinal horizontal cells is proposed [72-74]. In taste cells, ATP release through pannexin hemichannels is critical for communication to presynaptic cells [75,76]. If hemichannels made of Cx43 or related connexins release ATP, paracellular ATP could influence neighboring ORN activity as application of ATP to ORNs modulates neuronal responses [77]. In this study, the data from OlfDNCX mice do not allow me to discern efforts between gap junctions and hemichannels. Future studies will explore potential roles of hemichannels by continuously working with OlfDNCX mice.

### Disruption of gap junctions may affect odor activity maps

In OlfDNCX, regions in the glomerular layer of the olfactory bulb that were activated by octaldehyde ("domains" of activation) covered a smaller area than domains in WT (Figure 5). This observation indicates that electrical coupling through gap junctions results in activation of a larger number of glomeruli, implying that ORNs expressing different receptors (and hence targeting different glomeruli) are coupled through gap junctions. This inference agrees with my interpretation of the calcium imaging data presented here (Figure 4), with my interpretation of the implications of the EOG study in OlfDNCX mice (Figure 3), with our earlier immunohistochemical studies [23], with our modeling of electrically coupled ORNs [58], and with modeling by others [78]. The octaldehyde activity domains that were more active in WT than OlfDNCX were located in the ventromedial, medial and lateral areas of the glomerular layer adjacent to the domains with highest odor-evoked activity (Figure 5D). They receive axons from ORNs in ventral and lateral regions of the olfactory epithelium [79]. In normal mice, the olfactory epithelium in these regions expresses more Cx43 compared to dorsal recesses [23]. This topographical relationship between the areas of the olfactory bulb affected by disruption of gap junctions and the areas of olfactory epithelium expressing high amounts of Cx43 in WT mice further supports the premise that altered odor maps are associated with dysfunction of gap junctions in mature ORNs.

Several studies indicate that glomerular spatial activity patterns participate in encoding odor quality and intensity, and that the topography of activation is likely ruled by the topographical relationship between ORNs bearing a specific receptor and their glomeruli in the olfactory bulb [41,49,80-83]. In addition, studies on the electrical activity of neurons responding to particular odors in the olfactory bulb (and the antennal lobe in insects) suggest that synchronized oscillations may also be involved in coding for odor quality [84,85]. This study shows that impairment of gap junctions reduces olfactory sensitivity and affects the topography of odor-evoked activity in the glomerular layer of the olfactory bulb. The mechanism may contribute to the factor that odor-evoked sensory input generates long-lasting EPSP in mitral cells [6], as well as to the factor that glomeruli having high odor-evoked amplitude are those having relatively long response latency [5]. Because gap junctions in ORNs affect synchronous firing of ORNs and affect odor activity maps in the bulb, gap junctions in the olfactory epithelium are likely to play a role in odor detection and discrimination.

## Conclusions

This study has demonstrated for the first time that intercellular gap junctional communication between mature ORNs and other epithelial cells (ORNs and/or sustentacular cells) or hemichannel activity has functional implications in olfactory sensation under normal physiological conditions. I have provided evidence that gap junctional coupling modulates olfactory coding. The conclusion is drawn based on two independent studies: functional characterization of a dominant negative transgenic mouse OlfDNCX and calcium imaging to individual neurons situated in intact turbinates. Data from these studies demonstrate that gap junctional coupling is critical for maintaining olfactory sensitivity in subsets of olfactory neurons. Dysfunction of gap junctions in the peripheral pathway leading to reduced olfactory responses and altered odor maps indicates that gap junctions modulate quantitative and qualitative odor perception.

## Methods

### Generation of OlfDNCX transgenic mice

The transgene construct utilizes the OMP promoter to drive expression of Cx43/β-gal in mature ORNs (Figure 1). Dr. Cecilia Lo at the National Heart Lung and Blood Institute, National Institutes of Health provided us with the pEFZ vector used to produce their dominant negative transgenic mice [28]. The pEFZ vector uses the human elongation factor-1α promoter to drive expression of Cx43/β-gal gene. The Cx43/β-gal gene contains entire coding region of Cx43 and the Kozak consensus initiation sequence. The full length coding sequence of β-gal was directly appended in frame to the C-terminus of Cx43 by deleting the stop codon of Cx43 and the start codon of β-gal [28]. In this study, the human elongation factor-1α promoter was replaced by 880 bp of the proximal region of the OMP promoter (-829 to +51, where +1 is the transcription start site) (Figure 1) using HindIII and XbaI restriction sites. The fragment of the OMP promoter was obtained by PCR amplification of FVB mouse genomic DNA with the forward primer **CCCAAGCTTGGG**ATCTCTGTCTCCACCACTC and reverse primer **GCTCTAGAGC**CTACAGCGATTGCCACTG (added HindIII and XbaI restriction sites are shown in bold). Previous studies have shown that various sizes of OMP promoters are effective in driving expression of foreign genes in mature ORNs [86-88], suggesting that the OMP promoter driven Cx43/β-gal would be expressed only in mature ORNs in the olfactory epithelium.

To produce transgenic mice, the construct was linearized by removing back bone of the vector before injecting into fertilized FVB mouse oocytes. Perinuclear injection of the transgene yielded 32 offspring, four of which were positive for the transgene. However, only two survived to establish lines. There was no difference between experiments performed with the two transgenic lines, and all data presented in the manuscript are from the same transgenic line. Insertion of the transgene in the genome was confirmed by Southern analysis and PCR amplification of the region that bridges the proximal OMP promoter and Cx43 coding regions using the primer pair ATCTCTGTCTCCACCACTC and TTAGATCTCCAGGTCATCA. Mice in the study had mixed FVB and C57BL/6J background. Experimental animals were at least 8 wks old. All procedures were performed under protocols approved by the Animal Care and Use Committee of the University of Colorado at Denver and Animal Care and Use Committee of the Illinois Institute of Technology.

### Reverse transcription PCR

Total RNA from mouse turbinates was isolated with the TRIzol reagent according to the manufacturer's directions (Invitrogen, CA). Aliquots of 5 μg of total RNA were digested with RQ1 RNase-free DNase (Promega, WI) to eliminate genomic DNA contaminations. Digested RNA was divided into two groups for reverse transcription. In one group, SuperScript II (Invitrogen, CA) was omitted as the control. A unique region of cDNA complementary to mRNA encoding for Cx43/β-gal was amplified using a primer pair spanning the regions encoding for the two proteins fused in the construct (Cx43 and β-gal): TCCTGGGTACAAGCTGGTCACT and TAATTCGCGTCTGGCCTTCCTGT. The PCR product from olfactory turbinates was subcloned into pCRII (Invitrogen, CA) and sequenced.

### Real time quantitative PCR

qPCR was used to determine whether insertion of the dominant negative transgene induced overall changes of gene expression in the olfactory epithelium. Expression levels of a few gene transcripts in the olfactory epithelium were compared between WT and OlfDNCX using qPCR. Total RNA was extracted from mouse turbinates and digested with RQ1 RNase-free DNase as mentioned before. One-step qPCR was processed in the Research Technology Support Facility at Michigan State University with the primer pairs listed in Table 1. With exception of Cx43, all primers were designed to amplify fragments of coding regions (Table 1). Primer pairs of Cx43 anneal Cx43-3U to exclude transcripts of the transgene. For normalization, GAPDH gene was analyzed as an endogenous control. qPCR reactions were performed on an ABI 7700 real time PCR thermal cycler using SYBR Green master mix (Applied Biosystems, CA) under the following conditions: 48°C for 30·min, 95°C for 10·min, and 40 cycles of 95°C for 15·s followed by 60°C for 1·min. Each qPCR analysis was done in triplicate.

The Ct values of qPCR were analyzed using the comparative Ct (ΔΔCt) method described by the manufacturer. ΔCt values were calculated by normalizing Ct values to that of the endogenous control (GAPDH), and by subsequently calculating ΔΔCt values against the ΔCt value of a control mouse. Fold changes of gene expression for a particular gene between OlfDNCX and WT were then compared.

### *In situ *hybridization

The method was essentially the same as described in Zhang et al. [23]. Adult mice were anesthetized and perfused intracardially with 4% paraformaldehyde. Mouse snouts were then dissected, fixed overnight in 4% paraformaldehyde containing 25% sucrose, embedded in Tissue-Tek O.C.T. compound (Sakura Finetek, CA), and cut on a cryostat at -18°C. Tissue sections (12 μm) were stored at -80°C before use. Every 5th section was processed in each series.

A 369 bp DNA template corresponding to a portion of the β-gal coding sequence (nucleotides 2264 to 2632 in GenBank accession number U46491) was used to synthesize digoxigenin-labeled sense and antisense RNA probes. For *in situ *hybridization, tissue sections were brought to room temperature, treated with proteinase K (15 μg/ml in phosphate buffered saline (PBS)) for 5 min and post fixed for 15 min in 4% paraformaldehyde. Sections were rinsed in PBS three times for 10 min each prior to a 2 hr incubation in prehybridization solution (50% deionized formamide, 1× Denhart's solution, 750 mM sodium chloride, 25 mM ethylenediamine-tetraacetic acid (EDTA), 25 mM piperazine-N,N'-bis[2-ethane-sulfonic acid] (PIPES), pH 7.0, 0.25 mg/ml salmon sperm DNA, 0.25 mg/ml poly A acid and 0.2% SDS). Sections were then hybridized overnight with sense (as the control) or antisense digoxigenin-labeled RNA probes in hybridization solution (prehybridization solution containing 5% dextran sulfate) at 60°C. After hybridization, sections were washed three times for 10 min intervals in 2 × SSC/0.3% polyoxyethylene sorbitan monolaurate (Tween-20) followed by three washes in 0.2 × SSC/0.3% Tween-20 at 65°C. Detection of digoxigenin-labeled probes was based on the procedures suggested by the manufacturer (Roche, IN). The tissue sections were blocked for 2 hr in 10% sheep serum/2% bovine albumin/0.3% Tween-20. The sections were then incubated for 4 hr with alkaline phosphatase-conjugated anti-digoxigenin Fab fragments (1:1000 in blocking solution). Unbound Fab fragments were removed and the sections were incubated in nitroblue tetrazolium chloride and 5-bromo-4-chloro-3-indolyl phosphate substrate (NBT/BCIP). Control studies, using sense RNA as the probe, were performed under identical conditions each time.

### Western analysis

Mouse turbinates were homogenized in PBS and centrifuged at 2000 × g for 1 min in the presence of a cocktail of protease inhibitors (Sigma Cat No P8340). Aliquots of 60 μg protein were denatured and solubilized by boiling in SDS loading buffer (pH 6.8) and resolved in 8% polyacrylamide minigels. Western blots were performed according to Sambrook et al. [89]. Polyclonal rabbit anti-β-gal (ICN Pharmaceuticals, OH) and monoclonal mouse anti-Cx 43 (Chemicon International, CA) were used for western analysis.

### Immunohistochemistry

Immunofluorescence was used for the immunohistochemical localization of β-gal immunoreactivity. A primary antibody polyclonal rabbit anti-β-gal or guinea pig anti-β-gal (courtesy of Drs. Cindy Yee and Tom Finger) was visualized by indirect immunofluoresence with a secondary antibody linked to Rhodamine Red (Jackson ImmunoResearch Laboratories, PA). A confocal laser-scanning microscope (Olympus Fluoview) was used to examine the immunofluoresence. Incubations without primary antibodies resulted in no staining.

### Electrophysiology

Underwater EOG recordings were performed to examine olfactory responses to odors [90]. The decapitated mouse head was opened along the midline, and the endoturbinates were exposed by removing the septum (Figure 3A). Ringer's saline containing 145 mM NaCl, 5 mM KCl, 20 mM *N*-2-hydroxyethylpiperazine-*N*'-2-ethanesulfonic acid buffer (HEPES), 1 mM MaCl_2_, 1 mM CaCl_2_, 1 mM Na pyruvate and 5 mM D-glucose (pH 7.2) was perfused continuously over the surface of the turbinates. Saline and odorants were delivered by a glass capillary through a gravity-fed computer-controlled perfusion system with an approximate flow rate of 0.23 ml/s. Each odorant was presented 1 s for three times in 1 min intervals. The second response was used for analysis. Following stimulation with an odorant, the capillary was washed with saline for 2-3 min until EOG responses to saline were back to the basal level. Local field potential was recorded under current clamp using an Axopatch 200 B amplifier controlled by a PC computer with axon software (Clampex 8, Axon Instruments, CA). The recording electrode was filled with 0.9% agar made in Ringer's saline with 1% neutral red. The electrode was placed on the apical surface of endoturbinate IIb (Figure 3A), and the reference Ag/AgCl electrode was connected to bath saline. The recorded signals were low-pass-filtered at 20 Hz, digitized at 500 Hz and analyzed using the Axon software Clampfit. The EOG data were presented as normalized response magnitudes calculated as the magnitude of a test odorant divided by that of immediately preceding benzaldehyde. The data are shown as means and standard errors.

### Calcium imaging to the intact olfactory epithelium

The decapitated mouse head was opened along the midline, and the endoturbinates were exposed as described above. The olfactory bulb and bones around ectoturbinates were removed and the turbinates were loaded with fure-2 AM (Invitrogen, CA) similar to described before [91]. In brief, the turbinates were incubated in oxygen-saturated Ringer's saline containing 5 μM Ca^2+^-sensitive dye fura-2 AM and 160 μg/ml nonionic dispersing agent Pluronic F-127 at 37°C for 1 hr. They were mounted in a recording chamber with endoturbinates face up as shown in Figure 3A and continuously perfused with saline throughout the experiments. Ratiometric calcium imaging was performed at excitations of 340 nm (F340) and 380 nm (F380) in an Olympus upright microscope equipped with a 20×, 0.9 numerical aperture water immersion objective, a filter wheel (Sutter Instruments, Novato, CA), a 175w xenon lamp and a cooled CCD camera (SensiCam; Cooke Corporation, MI). Images were collected every 4 s using a data acquisition software Imaging Workbench 5.2 (Indec Biosystems, CA). The interval of stimuli was 15-40 s after the previous response returned to baseline. There was no waiting period immediately after BGA application because the study was to examine if gap junction uncoupling by BGA changes neuronal responses. Data were binned every four frames after recordings using the Excel program and presented as the ratio of F340/F380.

### Odor exposure and odor map analysis

Odor exposures prior to determination of *c-fos *expression were as described previously [49] with minor modification. Individual mice were placed in a 5 l glass jar and exposed to humidified fresh air for 40 min at 3.5 l/min and then exposed to octaldehyde delivered by the same fresh air 3 min at 5 min intervals over a 30 min period. The odor source was from a 47 mm diameter beaker equilibrated with 0.001% of octaldehyde diluted in odorless mineral oil.

Mice were sacrificed immediately after odor exposure, perfused with 4% paraformaldehyde, and the olfactory bulbs were harvested. Transverse sections (18 μm) of the olfactory bulbs were cut in a plane perpendicular to the olfactory tract [92]. Antisense cRNA transcribed from a mouse recombinant cDNA clone corresponding to positions 1842-1944 and 2061-2493 of the mouse *c-fos *gene (MUSFOS) was used to determine expression of *c-fos *mRNA in the juxtaglomerular cells surrounding glomeruli. Glomeruli were scored as positive when an arc of labeled juxtaglomerular cells spanning either 180° in any orientation or two 90° arcs spanning any region were identified.

The coordinates for each positive glomerulus are given in rostrocaudal distance and radial angle around a section where the anatomical landmarks served to determine the origins for the radial measurements [92]. The first section was defined by the point at which complete mitral cell and external plexiform layers can be identified. The 0-180° axis was drawn parallel to the ventral aspect of the subependymal layer. For the rostral sections, the origin was taken as one-third the distance from the dorsal to the ventral mitral cell layer. In sections containing the accessory olfactory bulb (AOB), the origin was defined as the point just ventral to the AOB. Posterior to the AOB, the origin was placed at the granular cusp. Activated glomerular locations in cylindrical coordinates were determined using a plugin for ImageJ and the data were then visualized as a color contour plot constructed in Microcal Origin without normalization. A point to point Mann-Whitney test was performed as in Schaefer et al. [49] to compare the difference of odor maps. A detail description of an upgraded odor mapping program is described elsewhere [48].

## Authors' contributions

CZ designed and performed the experiments.

## Authors' information

CZ is an assistant professor at the Illinois Institute of Technology. Her work on functions of gap junctions in olfactory sensation started a decade ago at the Rocky Mountain Taste and Smell Center, University of Colorado Denver.
